# *Myroides odoratimimus* urinary tract infection in an immunocompromised patient: an emerging multidrug-resistant micro-organism

**DOI:** 10.1186/s13756-018-0391-4

**Published:** 2018-08-06

**Authors:** Giovanni Lorenzin, Giorgio Piccinelli, Lucrezia Carlassara, Francesco Scolari, Francesca Caccuri, Arnaldo Caruso, Maria Antonia De Francesco

**Affiliations:** 1 Institute of Microbiology, Department of Molecular and Translational Medicine, University of Brescia-Spedali Civili, P. le Spedali Civili 1, 25123 Brescia, Italy; 2 Department of Nephrology, University of Brescia, Hospital of Montichiari, Brescia, Italy; 3 Institute of Microbiology and Virology, Department of Biomedical, Surgical and Dental Sciences, University of Milan, Milan, Italy

## Abstract

**Background:**

*Myroides* spp. are common environmental organisms and they can be isolated predominantly in water, soil, food and in sewage treatment plants. In the last two decades, an increasing number of infections such as urinary tract infections and skin and soft tissue infections, caused by these microorganisms has been reported. Selection of appropriate antibiotic therapy to treat the infections caused by *Myroides spp*. is difficult due to the production of a biofilm and the organism’s intrinsic resistance to many antibiotic classes.

**Case presentation:**

We report the case of a 69-year-old immunocompromised patient who presented with repeated episodes of macroscopic haematuria, from Northern Italy.

A midstream urine sample cultured a Gram negative rod in significant amounts (> 10^5^ colony-forming units (cfu)/mL), which was identified as *Myroides odoratimimus.* The patient was successfully treated with trimethoprim/sulfamethoxazole after antibiotic susceptibility testing confirmed its activity.

**Conclusion:**

This case underlines the emergence of multidrug resistant *Myroides* spp. which are ubiquitous in the environment and it demands that clinicians should be more mindful about the role played by atypical pathogens, which may harbour or express multidrug resistant characteristics, in immunocompromised patients or where there is a failure of empiric antimicrobial therapy.

## Background

*The Myroides* spp*.*, which were previously classified as *Flavobacterium* spp., are Gram negative, non-fermentative and non-motile bacteria. They do not traditionally belong to the normal human flora. *Myroides* genus includes two species: *Myroides odoratus* and *Myroides odoratimimus* [1]. They are considered low-grade opportunistic pathogens and are rarely isolated from clinical samples but, occasionally, they are life-threatening [2]. Due to the presence of flexirubin, they are yellow pigmented on culture and they are obligated aerobic rod bacteria with a characteristic fruity odour (strawberry-like) [2, 3].

Despite the low pathogenicity potential, managing *Myroides odoratimimus* is difficult because most strains are multi-drug resistant [4, 5]. In addition, *Myroides* has different virulence factors [5], has the capacity of co-aggregation and self-aggregation to form biofilm [6]. and possess a polysaccharide capsule, which makes the bacterial surface extremely hydrophobic.

## Case report

We present the case of a 69-year-old man with type II diabetes mellitus with ocular end-organ dysfunction, on oral hypoglycaemic agents, and with hypertension. He was also affected by an end stage renal failure requiring haemodialysis three times a week. Furthermore, he had other co-morbidities: ischaemic cardiomyopathy treated with oral anticoagulant therapy, mild chronic myelomonocytic leukemia (CMML), dyslipidemia and obesity.

In June 2016, a permanent urinary Foley’s catheter was positioned due to urinary retention.

In August 2017, the patient was seen to the emergency room (ER) of the Montichiari Hospital, Brescia, Italy. On admission, the patient was afebrile and upon physical examination, his vital signs (arterial pressure, heart rate and respiratory rate) were within normal limits. The patient gave a 3-day history of ongoing macroscopic haematuria and reported no lower urinary tract symptoms or other symptoms suggesting an inflammatory response or bleeding tendency. The patient had no history of abdominal or pelvic surgery. The international normalized ratio (INR) was 2.5 and hematologic parameters were within the normal range except red blood cell count, which was decreased (3 × 10 ^6^ /μL), related to kidney failure. Glycated haemoglobin (HbA1c) was 52 mmol/mol. Finally, he was discharged with a hemorrhagic cystitis diagnosis and he was empirically treated with ciprofloxacin at a renally-adjusted dose (250 mg 2/die for 1 week) with the complete resolution of the macroscopic heamaturia.

In September 2017, the patient was seen again to the ER for another episode of macro-hematuria. On admission, he had a temperature of 36.5 °C, the blood pressure and the heart rate were within the normal limits, and there weren’t relevant findings on physical examination; blood cultures were performed but they were negative. Glycated haemoglobin (HbA1c) was 39 mmol/mol.

The patient had already started at home ciprofloxacin (250 mg 2/die) independently, so the clinician suggested that he continued this therapy for 1 week.

In the same month, the patient underwent a full urological investigation of haematuria, to exclude cancer or other abnormalities, a transrectal ultrasound, which identified a benign prostate adenoma, a cystoscopy which was negative for neoplasia, and a urinary cytology screening, which was negative for malignant cells. Despite the antibiotic therapy, the patient had symptoms related to urinary tract infection: bladder tenderness, hematuria and pelvic discomfort.

Therefore, a urine sample for culture was obtained by removing the indwelling catheter and obtaining a midstream specimen analysed by the laboratory of Microbiology and Virology of the Spedali Civili Hospital, Brescia, Italy; then, a new Foley’s catheter has been replaced. Urinalysis showed the presence of nitrites, leukocyte esterase and 6–7 leukocytes per high power field by microscopy.

Patient was discharged with clear instructions given to him for a proper care of the urinary catheter and for a correct hand hygiene to prevent infections, and the prescription of an empirical antibiotic therapy. It comprised levofloxacin at 250 mg for 10 days, switched then to amikacin 500 mg intravenously for the following three dialysis sessions (for a total of one week) for cover against multi-drug resistant *Pseudomonas aeruginosa*, as guided by local epidemiology.

The urine culture grew a > 10^5^ colony-forming units (cfu)/ml of a gram-negative rod. The bacterium was isolated from Columbia CNA agar (BioMérieux, Florence, Italy) after 24 h of incubation in aerobic conditions. The colonies appeared round, mucoid, yellow pigmented and with a fruity smell. The initial identification as *Myroides spp*. was performed using a matrix-assisted laser desorption ionization-time of flight mass spectrometry (MALDI-TOF MS) according to the manufacturer’s instructions. The definitive identification was obtained with 16SrRNA gene sequencing. The obtained sequence was compared with the sequences in the GenBank database (http://ncbi.nlm.gov/blast) and it exhibited a 100% identity homology with *Myroides odoratimimus* strain BK21.

The antimicrobial susceptibility testing (AST) was first performed by using the standard disc diffusion on Mueller-Hinton agar. Then, the minimum inhibitory concentrations (MICs) were determined by automated microdilution broth test (BD-Phoenix NMIC-502, Becton Dickinson, Milan, Italy). The minimum inhibitory concentrations (MICs) were confirmed by Etest (BioMérieux). Since the breakpoints for *Myroides* spp. were unavailable, the interpretation of the results was performed according to the EUCAST guidelines for non-species related PK-PD breakpoints. The isolated strain was resistant to all beta-lactams, with and without inhibitors (Piperacillin/Tazobactam, MIC = 64; Ticarcillin/Clavulanate, MIC = 128; Ceftazidime/Avibactam, MIC = 32; Imipenem, MIC = 8; Meropenem, MIC = 4) and it was also resistant to fluoroquinolones, aminoglycosides, fosfomycin, nitrofurantoin and polymyxin. This conferred to the isolated strain a multi-drug resistance pattern. This strain was susceptible only to trimethoprim /sulfamethoxazole with a MIC of 1/19. A test was performed to assess beta-lactamase and carbapenemase production (ROSCO diagnostics, Biolife, Milan, Italy) according to the manufacturer’s instructions. The results showed the absence of synergy between the meropenem disk and the dipicolinic acid, the phenylboronic acid, the EDTA and the cloxacillin.

In October 2017, due to the inadequacy of empiric therapy, the patient suffered from another hemorrhagic cystitis episode. Another urine culture confirmed the presence of a multi-drug resistant *Myroides odoratimimus* strain. According to the antibiotic susceptibility results, the patient was treated with trimethoprim/sulfamethoxazole at a renally-adjusted dose (160/800 mg daily for 2 weeks) which led to the resolution of macroscopic haematuria. In addition, in the same month, in order to reduce the possibility of recurrent UTIs, the urinary catheter was definitively removed.

**Fig. 1:**
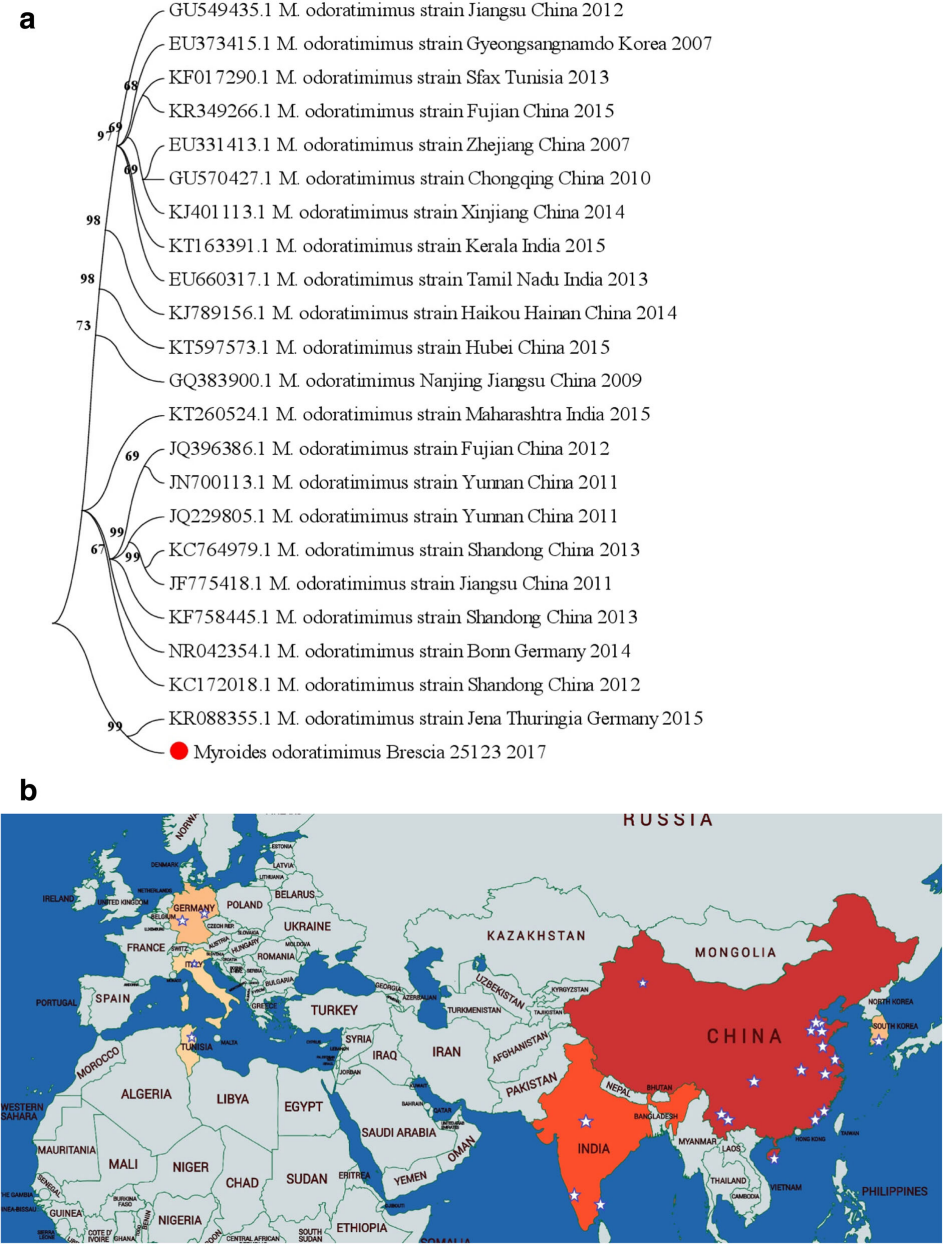
Geographical analysis (**a**) and phylogenetic analysis (**b**) based on *Myroides odoratimumus* 16SrRNA gene sequences. The strain from the immunocompromised patient in Italy is indicated with the red point. Reference strains from GenBank with their accession number are shown. The tree was constructed by the neighbor-joining method based on Kimura’s two-parameter model distance matrices with the MEGA program (version 7.0.26). Branch values are shown in the figure

Then, we tested its ability to grow in the form of biofilm. A Crystal Violet assay (CV) was performed to evaluate the production of biofilm at different concentrations of glucose and it was measured by spectrophotometry (NanoDrop™ Spectrophotometer, Thermo Fisher). The results indicated that this strain could be classified as a “strong biofilm-producer” [7], which is able to produce a high amount of biofilm when it is compared to the reference strains (*Pseudomonas aeruginosa* PAO1, strain ATCC 15692). The increase of glucose concentration facilitates the production of biofilm by *Myroides odoratimimus,* contributing to an increase in vivo of its virulence. Therefore, a strong biofilm-producing bacteria, like *Myroides odoratimimus,* is well protected against antibiotics. A phylogenetic analysis was performed using the Quick Bioinformatics Phylogeny of Prokaryotes web-server and the data were then re-analysed using the Molecular Evolutionary Genetics Analysis software (MEGA 7.0.26) [8]. Geographical phylogeny was then extrapolated from the Gene-Bank database with a self-written programme. The results showed that our strain clustered with a strain isolated in Jena, Germany (Fig. 1a). The geographic analysis showed that this pathogen is poorly represented in Western Europe (Fig. 1b).

## Discussion

Nowadays, the range of community and hospital acquired infections caused by atypical pathogens is continuously being updated. This increase in the number of newly described microorganisms is due to the use of both molecular identification, such as 16S rRNA sequencing and to the introduction in clinical microbiology laboratories of matrix-assisted laser desorption ionization–time of flight (MALDI-TOF) mass spectrometry.

The emergence of these microorganisms is associated with and impacted on by infection control and antimicrobial stewardship.

The antimicrobial resistance (AMR) has reached alarming levels in different parts of the world. As a result, many available treatment options are becoming ineffective. The major concern in AMR is the dissemination of bacteria with resistance to several antibiotics, also known as “superbugs”.

The inappropriate, and often uncontrolled, use of antibiotics has led to a global AMR epidemic, as it is defined today.

Current antibiotic use in great amounts in humans and animals and subsequent release of antibiotic residues in the environment give rise to a selection pressure that leads to the increase in antibiotic resistant bacteria. In fact, once ingested, most antibiotics are eliminated not metabolized. They can move through sewage systems or directly into water and soil, and mix with environmental bacteria adding pressure for selection of antibiotic resistant organisms. Human exposure to environmental bacteria can occur through drinking water, eating food or by direct contact with the environment.

*Myroides* spp. can be classified as a multi-drug resistant environmental organism and can harbour different resistance mechanisms simultaneously, as demonstrated in this paper and in other studies [9]. Intrinsic resistance to β-lactamases is due to the presence of two metallo-β-lactamases, MUS-1 and TUS-1, which share a 73% of amino acid identity [4]. Furthermore, a resistance island was found on the chromosome of the bacterium [10]. This region has different types of resistance genes, including tetX (conferring tetracycline resistance), cat (chloramphenicol resistance) and bla-OXA-347 and bla-OXA-209 (conferring β-lactam resistance).

Moreover, it has been recently found that *Myroides odoratimimus* not only have common virulence factors, like *bauE* gene to acquire iron competing with the host and adherence factors (*DnaK, Hsp60*), but also can survive intracellularly (*katA, clpP, EF-Tu, and sodB*), even in human stomach (*ureA, ureB, ureG*), can disseminate easily and is able to destroy human tissues [5].

In addition, our strain is a strong biofilm producer. Biofilms are the sessile bacterial communities which adhere to both biotic and abiotic surfaces, such as medical devices. The bacteria are entrapped within a self-produced extracellular polymeric matrix [11]. Biofilm formation is an important virulence factor for many pathogens; in fact, it has become obvious that sessile bacterial cells in the biofilms express properties which are different from the properties of planktonic cells, for example, the ability to escape host defense, but also the higher resistance to antibacterial agents [12, 13]. The production of a strong biofilm is a serious problem because it increases pathogenicity in device-related infections and it is often associated with therapeutic failure, as well as persistence of infections [14]. The development of biofilm by *Myroides spp* can be of significant health hazard often leading to recurrent infections, as demonstrated in this paper and in other studies [6].

Our isolate was resistant to all the tested antibiotics except trimethoprim/sulfamethoxazole. The empirical therapy with fluoroquinolones and aminoglycoside was unsuccessful. The resistance observed might be due to an uncontrolled and excessive use of these drugs, in particular fluoroquinolones, which are used, when empirical clinical measures are required after taking urine samples for analysis and culture, as the first-choice drugs in treatment of patients with complicated UTI, according to the European guidelines [15].

In our patient, different risk factors played an important role in causing a multi-drug resistant *Myroides* urinary infection, such as the presence of prolonged urinary catheterisation and an immunocompromised condition. Repeated hospital admissions of the patient might represent an independent risk factor for colonization and infection with multi-resistant microorganisms such as *Myroides spp*. Outbreaks of UTIs due to *Myroides odoratimimus* and hospital acquired are already reported [16–18].

In this case, the source of the infection has not been determined, but our hypothesis is that the patient may have acquired the infection from an environmental source, maybe related to poor hand hygiene during the catheter care.

In literature there are several cases which associate *Myroides* spp. with different types of infections such as soft tissue infections [6, 19], UTI [16–18], sepsis [2, 20], bacteremia [21, 22], cellulitis [23, 24], pericardial effusion [25], pediatric severe burn injury [26], fulminant erysipelas [27] and urosepsis [28]. Many of these case reports were from India, Turkey, Syria Tunisia, Belgium, Italy and Greece. The phylogenetic analysis showed a small cluster of our strain with a European isolate.

## Conclusions

Clinicians should be aware of atypical pathogens, in particular, in immunocompromised population, and urine culture should be considered at an earlier stage in these kind of patients due to the presence of less virulent organisms that may be harbouring important resistance mechanisms.

A well-designed antimicrobial stewardship associated with an efficient infection control are essential to limit the spread of these new emerging pathogens.

## Abbreviations

CA-UTI: Catheter associated urinary tract infection
CNA: Columbia nalidixic acid
MIC: Minimal inhibitory concentration
PK-PD: Pharmacokinetic-pharmacodynamic
